# Closer Look at Inverse Electron Demand Diels–Alder and Nucleophilic Addition Reactions on *s*-Tetrazines Using Enhanced Sampling Methods

**DOI:** 10.1007/s11244-021-01516-y

**Published:** 2021-10-23

**Authors:** Rangsiman Ketkaew, Fabrizio Creazzo, Sandra Luber

**Affiliations:** Department of Chemistry, University of Zurich, Winterthurerstrasse 190, 8057 Zürich, Switzerland

**Keywords:** iEDDA reaction, Azaphilic addition, Metadynamics, Blue moon ensemble

## Abstract

Inverse electron demand [4+2] Diels–Alder (iEDDA) reactions as well as unprecedented nucleophilic (azaphilic) additions of R-substituted silyl-enol ethers (where R is Phenyl, Methyl, and Hydrogen) to 1,2,4,5-tetrazine (*s*-tetrazine) catalyzed by $\hbox {BF}_{3}$ have recently been discovered (Simon et al. in Org Lett 23(7):2426–2430, 2021), where static calculations were employed for calculation of activation energies. In order to have a more realistic dynamic description of these reactions in explicit solution at ambient conditions, in this work we use a semiempirical tight-binding method combined with enhanced sampling techniques to calculate free energy surfaces (FESs) of the iEDDA and azaphilic addition reactions. Relevant products of not only *s*-tetrazine but also its derivatives such as $\hbox {BF}_{3}$-mediated *s*-tetrazine adducts are investigated. We reconstruct the FESs of the iEDDA and azaphilic addition reactions using metadynamics and blue moon ensemble, and compare the ability of different collective variables (CVs) including bond distances, Social PeRmutation INvarianT (SPRINT) coordinates, and path-CV to describe the reaction pathway. We find that when a bulky Phenyl is used as a substituent at the dienophile the azaphilic addition is preferred over the iEDDA reaction. In addition, we also investigate the effect of $\hbox {BF}_{3}$ in the diene and steric hindrance in the dienophile on the competition between the iEDDA and azaphilic addition reactions, providing chemical insight for reaction design.

**Supplementary Information:**

The online version contains supplementary material available at 10.1007/s11244-021-01516-y.

## Introduction

A growing number of studies about the Diels–Alder (DA) reaction has demonstrated in recent years its prevalence in both traditional and modern organic chemistry, bioinorganic and bioorthogonal chemistry. Among all DA reactions investigated to date, the [4+2] cycloaddition of 1,2,4,5-tetrazines (*s*-tetrazines) and various dienophiles, referred to as inverse electron demand DA (iEDDA) reaction, is the one that satisfies most bioorthogonal criteria (such as fast, selective, biocompatible and catalyst-free [1, 2]), which are necessary for the use in various applications ranging from protein labeling to cancer imaging or materials science [3].

It has been well known that the DA reaction generally occurs as a major outcome when *s*-tetrazine reacts with common dienophiles. However, our recent work in close collaboration with the Gademann group reported an unprecedented nucleophilic addition reaction of 3-bromosubstituted *s*-tetrazine (3-Br-*s*-tetrazine) which is in contrast to [4+2] cycloaddition reactions [4]. Moreover, the direct nucleophilic attack to nitrogen in *s*-tetrazine has rarely been described in the literature and is generally restricted to 1,2,4,5-tetrazine and other tetrazine derivatives such as 1,2,3,4-tetrazine and 1,2,3,5-tetrazine [5–7]. The investigation performed by our experimental collaborators revealed a surprising discovery in reactivity of 3-monosubstituted *s*-tetrazines, a small *s*-tetrazine building block, with silyl-enol ethers for the synthesis of e.g. macromolecules [4]. In this work, we, therefore, focus on chemical competition between [4+2] cycloadditions and azaphilic addition reactions of 3-Br-*s*-tetrazine and silyl-enol ethers. In addition, one main challenge is to observe the aforementioned reactions and their dynamics at a molecular level in the experiments since, e.g. most of the single-step reactions occur very fast which makes it difficult to catch up with the desired products. To address this issue, computational simulations can be used to provide insight by mimicking the chemistry and allowing additional information about processes at the molecular level behind the iEDDA and azaphilic additions of *s*-tetrazines, providing meaningful details not detectable with only experimental techniques.

Usually the polar effect of the substituent and the stabilization of valence electrons in the *s*-tetrazine control the type of DA reaction. It has been recognized that *s*-tetrazines are good candidates as dienes for the iEDDA reaction. Moreover, a Lewis acid can be used as a catalyst to activate the *s*-tetrazine by lowering the level of its lowest unoccupied molecular orbital (LUMO), for which corresponding iEDDA reactions with electron-rich dienophiles could occur under relatively mild conditions and arrive at the desired product in the form of a bridged structure [8, 9]. A number of Lewis acids, e.g. $\hbox {SO}_{3}$, $\hbox {Br}_{2}$, and $\hbox {AlCl}_{3}$, have been used. In particular, boron halides possess low-lying orbitals that are suitable for the cycloaddition reaction. The electrophilicity of boron in Lewis acids increases in the order of $\hbox {BBr}_{3}$ $\rightarrow$ $\hbox {BCl}_{3}$ $\rightarrow$ $\hbox {BF}_{3}$. Hence it has been proved by several calculations [10–12] and experiments [13, 14] that $\hbox {BF}_{3}$ is a highly efficient catalyst for the iEDDA reactions [1]. At the same time, based on experimental results reported in the previous study, the reaction could produce the product with up to 82% yield in the presence of $\hbox {BF}_{3}$ [4]. In addition, the $\hbox {BF}_{3}$ Lewis acid bound to the diene can indirectly influence its electronic state via electron delocalization through the N-containing aromatic ring [15]. Therefore, it has also been important to investigate the effect of $\hbox {BF}_{3}$ on chemical selectivity of which different positions of nitrogen atoms on the *s*-tetrazine ring are compared [16, 17].

From a computational point of view, models have been developed over decades to mimic chemical reactions and capture physical/chemical changes at the atomic level. Various reactions were used as example tests for the developed method’s ability evaluation. The DA reaction is one of the popular choices as it can take place via concerted or stepwise mechanisms, depending on the experimental conditions. Both static Density functional theory (DFT) and (classical) molecular dynamics (MD) calculations were performed to investigate the dynamically concerted mechanism of the DA reaction [18–22]. For the unusual regio- and stereo-selectivity of DA reactions, selected works related to the inverse DA reaction are mentioned in the following. Shrivastav et al. studied the reaction energetics and kinetics of 2-pyrone complex undergoing the retro DA reaction [23]. They also calculated the free energy surface (FES) of the DA reaction using Car-Parrinello molecular dynamics with MetaD to obtain the Gibbs activation energy. Li et al. used quantum mechanics/molecular mechanics and Multistate Bennett Acceptance Ratio and Weighted Thermodynamic Perturbation methods to calculate the FES of a similar DA reaction [24]. These notable studies warrant that the FES is a crucial descriptor to shed light on theoretical explanation of the DA reaction.

A growing number of significant developments of simulation techniques to calculate the FES have been presented, in particular enhanced sampling techniques, which have been integral to many discoveries in chemistry and provide attractive tools including umbrella sampling [25], MetaD [26–28] and Blue Moon (BM) ensemble [29]. Previous studies addressed by the MetaD method have proven a wide range of applications in chemical reactions which can be described by reaction coordinates or the so-called collective variables (CVs). The CVs have to be chosen in such a way that various conformations can be distinguished and the validity of the reaction pathways can be demonstrated by the FES.

**Fig. 1:**
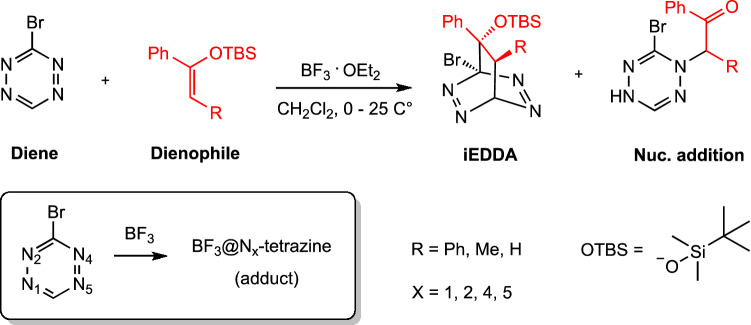
Inverse electron demand Diels–Alder (iEDDA) and nucleophilic (azaphilic) addition reactions. A schematic reaction in the box shows the $\hbox {BF}_{3}$-mediated *s*-tetrazine adduct. $\hbox {BF}_{3}$@N$_X$ denotes binding of $\hbox {BF}_{3}$ at the X position of nitrogen atom in the ring

The aim of this work is to characterize the kinetics and the thermodynamics behind the iEDDA and azaphilic additions of 3-Br-*s*-tetrazine (with and without $\hbox {BF}_{3}$) and silyl-enol ethers (as dienophile) with R substituent group; where R is Phenyl (**Ph**), Methyl (**Me**), and Hydrogen (**H**) (see Fig. 1), providing unprecedented free-energy profiles by means of enhanced sampling-based MetaD and BM ensemble simulations. DFT-based molecular dynamics (DFT-MD) has been employed for a wide range of applications ranging from biological systems, over material design, to molecular chemical modeling [30–34]. However, DFT-MD can become computationally demanding, depending, among others, on the size of the modeled system and process to be studied. In order to speed up our simulations, we adopt a tight-binding (TB) method [35, 36], namely Geometry, Frequency, Noncovalent, eXtended TB (GFN-xTB) based on already defined semi-empirical parameters which allow to reduce the computational time required (in comparison with standard DFT-MD algorithms). The use of GFN-xTB has been gradually growing with a wide range of applications for thermodynamics calculations [37, 38], transition metal complexes [39, 40], and spectroscopy [41–44]. The aim of this work is to complement our previous studies by the inclusion of finite temperature effects, explicit solvent, and the effect of substituents on the dienophile and to provide additional computational insight into the studied systems as well as the role of $\hbox {BF}_{3}$.

In the following sections, we first give an overview of WT-MetaD and BM methods that we used for performing the density functional tight binding MD (DFTB-MD) simulations. A clear and meaningful discussion on the selection of CVs for WT-MetaD and BM is also accommodated in the study of the FES. In this work, not only standard CVs are employed, but we also utilize recently developed adjacent-contact-matrix-based atomic coordinate functions including Social PeRmutation INvarianT (SPRINT) coordinates and path nuclear collective variable (path-CV), which have been successfully adopted in Ref. [45] on the formation of fullerene from graphene and in Ref. [46] on methanol oxidation.

The remainder of the paper proceeds with the structural analysis in order to assess the conformation change of reaction partners along WT-MetaD simulations. We focus on azaphilic addition reactions and show that the 3-Br-*s*-tetrazines are stereoselective to the replacement of substituent (R group) in the silyl-enol ether. Moreover, the direct attack of $\hbox {BF}_{3}$ to different positions of nitrogen in 3-Br-*s*-tetrazine plays an important role in the energetics of the reaction, leading to chemical selection between normal iEDDA and nucleophilic addition reactions.

## Methods

### Computational Details

We use a cubic box with a side length of 15 ${\text{\AA} }$ size containing one DA product and 31 dichloromethane (DCM) molecules in order to mimic the experimental density of 1.33 $g/cm^3$. Periodic boundary conditions (PBCs) are applied in all three spatial directions. The box size and number of molecules were chosen as the best compromise between the computational complexity of the system and the reliability of the calculations in reproducing the expected physical/chemical properties. For the DFTB-MD simulations, the GFN-xTB [35] method based on semiempirical parameters in the CP2K package version 7.1 [47] was used. The GFN-xTB basis set was employed with an auxiliary plane-wave basis set using a cutoff of 800 Ry. For the DFT-MD simulation, the atoms were instead described by the DZVP-MOLOPT-SR-GTH basis set and GTH-PBE pseudopotentials [48–50]. The Perdew–Burke–Ernzerhof (PBE) exchange-correlation functional [51] and Grimme’s D3 dispersion correction [52] were employed.

All systems containing the product of either iEDDA or azaphilic addition reaction were equilibrated using isothermal-isobaric (NPT) followed by canonical (NVT) ensembles with Nose-Hoover thermostat using a time constant of 100 fs using an MD time step of 0.5 fs at a constant temperature of around 300 K. We note here that, for NPT ensemble, in addition to the thermostat, a barostat is used with the pressure set to 1 Bar and a time constant of 80 fs. Unbiased MD simulations were carried out for 25 ps, whereas biased simulations were carried out for 50 ps. PLUMED version 2.6 [53] was used as a plugin in conjunction with QUICKSTEP [54] in CP2K for driving WT-MetaD simulations and reconstructing the FES. Helmholtz free energies of iEDDA and azaphilic addition reactions were computed.

All static calculations were carried out by means of DFT using TURBOMOLE version 7.3 [55]. For DFT calculations, we adopted computational parameters tested and used in the previous work [4]. The structures were optimized using BP86 [56, 57] with a def2-TZVP [58] basis set. Single-point (SP) energies were computed using B3LYP [59] at the same level of basis set. In addition, geometry optimization and SP energy calculations were also performed with PBE functional and def2-SVP basis set. Grimme’s 3D dispersion correction was employed. The solvent effects of DCM were considered using the COSMO model [60] (dielectric constant = 8.93) in TURBOMOLE. Vibrational frequency calculations were carried out to verify the TS and obtain Gibbs free energies.

### Metadynamics

MetaD is developed based on a history-dependent Gaussian bias potential using the so-called CV ($\xi$) which usually relies on nuclear coordinates. For the sake of brevity, we do not explicitly note here the dependence of $\xi$ on e.g. nuclear coordinates. Within a standard MetaD method, the sum of Gaussian potentials deposited along the reaction coordinate and simulation time is given as

1 $$\begin{aligned} V(\xi ,t) = \sum ^{t' < t}_{t' = \tau , 2\tau , \ldots} W~exp\Bigg (-\sum _{i=1}^{d}\frac{(\xi _i-\xi _i(t'))^2}{2\sigma _i^2} \Bigg ) \end{aligned}$$

where $\xi _i$ is the value of the *i*-th CV at time t, $\xi _i(t')$ at time $t'$, *d* is the number of CVs, *W* the height, $\tau$ the deposition stride, and $\sigma _i$ the width of the Gaussian potential used for the *i*-th CV. In standard MetaD, Gaussian bias potential with constant *W* is accumulated during the entire simulation. This might lead to overfilling of the configurational space.

To avoid this shortcoming, well-tempered metadynamics (WT-MetaD) developed by Barducci et al. has been introduced with a slow-converged potential at the final state of the calculation [61]. The height of the Gaussian potential is controlled with decreasing simulation time

2 $$\begin{aligned} W = W_{0} ~ exp(-\frac{V(\xi ,t)}{k_{\text {B}}\varDelta T}) \end{aligned}$$

where $W_0$ is the initial height of the Gaussians potential and $\varDelta T$ is a parameter specifying rescaling rate with the dimension of temperature. To perform WT-MetaD simulations with the PLUMED package, the so-called “bias factor” $\gamma$ is chosen which is defined as follows:

3 $$\begin{aligned} \gamma = \frac{T + \varDelta T}{T} \end{aligned}$$

where $T + \varDelta T$ is the temperature of the CVs and *T* is the system temperature. When choosing $\gamma$ care has to be taken to ensure that the free energy barriers can be efficiently crossed in the appropriate simulation time scale [53].

Once the metadynamics simulation has converged at a longer time ($t\rightarrow \infty$), free energy *F* as a function of $\xi$ can then be analytically computed by integrating Eq. 1,

4 $$F(\xi ) = - V(\xi ,t\rightarrow \infty )$$

### Blue Moon Ensemble

In the BM ensemble, the FES is calculated as a function of the degree of freedom of a given reaction coordinate $\xi ' \equiv \xi '(\{r_{}\})$, which in general depends on a subset of *N* nuclear positions $\mathbf{r}_{\mathrm{i}}$. Integration of average forces $F_{\xi^{\prime}}$ results in the free energy profile given by

5 $$\varDelta F(\xi ',T) ~=~ -\int _{\xi _0}^{\xi _1} F_{\xi^{\prime}} d{\xi^{\prime}}.$$

The average force $F_{\xi^{\prime}}$ is derived from the Lagrange multiplier $\lambda$ according to

6 $$F_{\xi^{\prime}} ~=~ \frac{\langle Z^{-1/2}[\lambda - k_{\text {B}}TG]\rangle _{\xi^{\prime}}}{\langle Z^{-1/2} \rangle _{\xi^{\prime}}}$$

for which $k_{\text {B}}$ is the Boltzmann constant. The remaining terms, *Z* and *G*, are the so-called “Fixman mass-metric tensor correction” and correction factors associated with the transformation from generalized constraints to Cartesian coordinates, respectively. They are defined as

7 $$\begin{aligned} Z = \sum _{i=1}^{N} \frac{1}{m_i} \left( \frac{\partial \xi }{\partial \mathbf {r}_{\varvec{i}}}\right) ^2 \end{aligned}$$

and

8 $$\begin{aligned} G = \frac{1}{Z^2} \sum _{i=1}^{N} \sum _{j=1}^{N} \frac{1}{m_i}\frac{1}{m_j} \frac{\partial \xi }{\partial \mathbf {r}_{\varvec{i}}} \cdot \frac{\partial ^2 \xi }{\partial \mathbf {r}_{\varvec{i}} \partial \mathbf {r}_{\varvec{j}}} \cdot \frac{\partial \xi }{\partial \mathbf {r}_{\varvec{j}}} \end{aligned}$$

where $m_i$ is the atomic mass of nucleus *i*, and the corresponding position vector is $\mathbf {r}_{\varvec{i}}$[62]. When the bond distance $r_{ij}$ is chosen as CV, both *Z* and *G* become constant values [63]. Therefore, Eq. 6 can be rewritten as

9 $$f_{\xi^{\prime}}~=\langle \lambda \rangle _{\xi^{\prime}}.$$

In all other cases where the order parameter involves multiple distances, *Z* and *G* have to be derived analytically and evaluated for each step of the simulation.

## Results and Discussion

### Studied System

**Fig. 2:**
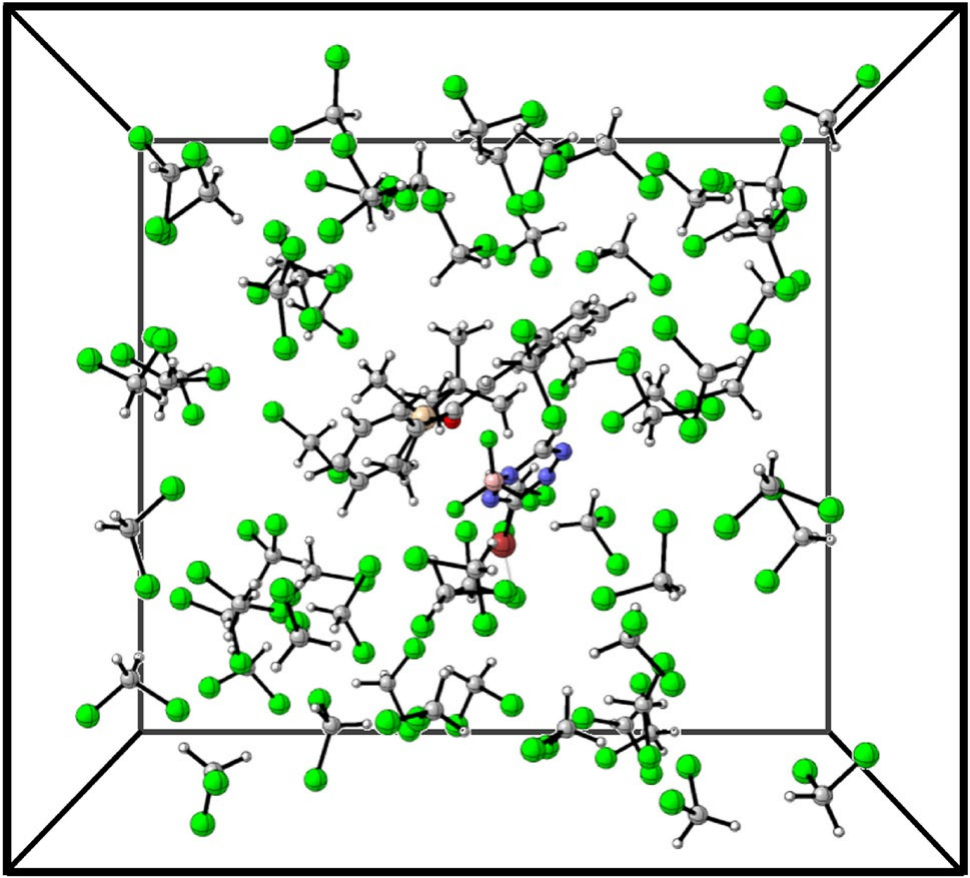
The periodic simulation box containing silyl-enol ether with Phenyl substituent group, 3-Br-*s*-tetrazine, and 31 DCM molecules

We studied the DA reactions of 3-Br-*s*-tetrazine and silyl-enol ether with R substituents (where R is Phenyl, Methyl, or Hydrogen) in DCM solvent. Systematic evaluation of the system in equilibrium resulting in the appropriate steady-state size of a cubic simulation box with side length of 15 ${\text{\AA} }$ side has been performed using DFTB-MD with the GFN-xTB method (see Fig. 2). We tested our computational setup calculating common chemical and physical parameters of the pure DCM system for which the data are reported in Table 1. Those primary data include density $\rho$, enthalpy of vaporization $H_{vap}$, and viscosity $\eta$. More information regarding $\varDelta H_{vap}$ and $\eta$ can be found in the Supporting Information (SI). We note that the energy related to the association of the two reaction partners and corresponding reorganization of the solvent is not considered in this work.

**Table 1:** Comparison of the observables for a pure DCM system experimentally measured or calculated at 298 K. The computed properties include density $\rho$, the change of enthalpy of vaporization $\varDelta H_{vap}$, viscosity $\eta$, and radial distribution function *g*(*r*) for the C–C pair. The computational results in this work were obtained from the NVT ensemble simulation. We note that the values obtained from previous work were computed by a classical MD simulation in NVT ensemble

Property	DFT-MD	DFTB-MD/GFN-xTB	Prev. Work [64]	Expmt. [65]
$\rho$ (g/cm^3^)	1.320	1.322	1.321	1.327
$\varDelta H_{\mathrm{vap}}$ (kcal/mol)	7.86	8.15	$8.17 \pm 0.04$	8.20
$\eta$ ($10^{-5}\ \hbox {cm}_{2}$/s)	3.6	3.2	$3.4 \pm 0.2$	$3.9 \pm 0.6$
$g(r) ({\text{\AA} })$	4.85	4.72	–	4.80[66]

Further calculations of radial distribution functions (*g*(*r*)) of pure DCM were carried out and they are available in the SI. The *g*(*r*) by GFN-xTB indicates that the configuration distribution of molecules for the C–C pair has a peak around 4.72 ${\text{\AA} }$, while the one of the Cl–Cl pair is around 3.05 ${\text{\AA} }$. These findings are in good agreement with those by DFT-MD: 4.85 ${\text{\AA} }$ for the C–C pair and 3.39 ${\text{\AA} }$ for the Cl–Cl pair, and experimental *g*(*r*) of 4.80 ${\text{\AA} }$ for the C–C pair of the same system.

### Choosing Collective Variables

We use the standard WT-MetaD for one-dimensional and two-dimensional FES simulations (parallel-bias WT-MetaD [67]) to enhance the crossing of high energy barriers for iEDDA and azaphilic addition reactions. Choosing CVs matters the simulation as they characterize the occurrence of long-lived metastable states [68]. That is, CVs have to be defined as a physical change parameter that drives the reaction coordinates or region where the reaction takes place. Moreover, the identification of appropriate set of CVs is largely dependent on and specific to the system.

**Fig. 3:**
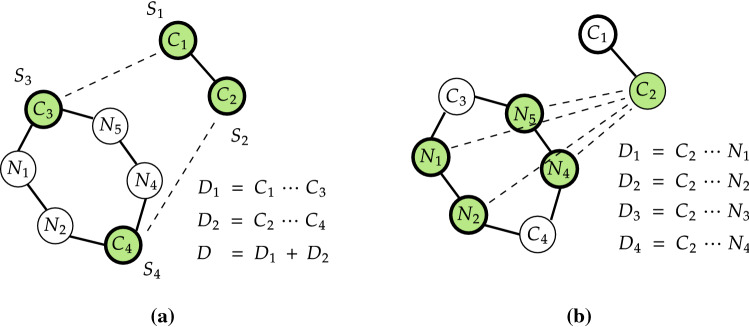
Outlines of **a** DA and **b** azaphilic additions with the different CVs sets. **a** $D_{1}$ and $D_{2}$ refer to specific $C_{1} \cdots C_{3}$ and $C_{2} \cdots C_{4}$ bond distances of the reaction (used as CVs), respectively. $S_{i}$ coordinates are computed and sorted from chosen four carbon atoms: $C_{1}$, $C_{2}$, $C_{3}$, and $C_{4}$, resulting in $S_{1}$, $S_{2}$, $S_{3}$, and $S_{4}$. (b) $D_{i}$ refers to specific bond distance between $C_{2}$ and i-*th* N atom. We note here that for the C-N bond distance of the azaphilic addition there are only two SPRINT coordinates used. Other atoms are not shown for the sake of simplicity

In our case, both iEDDA and azaphilic additions are characterized by concerted mechanisms in which bond forming and bond breaking take place in a single step [8], therefore the most straightforward way is to use the bond distance between 3-Br-*s*-tetrazine and silyl-enol ether as CV. As shown in Fig. 3, bond distances ($d_{1}$ and $d_{2}$) and the sum of the two bond distances ($D = d_{1} + d_{2}$) are straightforward CVs to represent the (time) evolution of the reaction, and are used to perform two- and one-dimensional WT-MetaD simulations, respectively. We also use the SPRINT coordinate [69] given by

10 $$S_{i} = \sqrt{N}\lambda ^{max} \nu _{i}^{max,sorted}$$

with

11 $$\nu _{i}^{max} = \frac{1}{(\lambda ^{max})^M}\sum _{j}a_{ij}^{M} \nu _{j}^{max}$$

$\lambda ^{max}$ and $\varvec{\nu }^{max}$ are respectively the maximum modulus eigenvalue and the corresponding eigenvector of adjacency matrix ($\varvec{A}$) with relation values between two atoms *i* and *j* of the system containing *N* atoms. Superscript *sorted* indicates that $\varvec{\nu }^{max}$ is sorted in ascending order where identical atom types are combined together into one value. $a^{M}_{ij}$ is the number of walks of length *M* connecting atoms *i* and *j* [69]. In this work we set *M* to be 1 [69]. The $a_{ij}$ element of $\varvec{A}$ is defined by the following switching function

12 $$\begin{aligned} a_{ij} = \dfrac{\left( 1 - \dfrac{r_{ij}}{r_{0}} \right) ^{n}}{{\left( 1 - \dfrac{r_{ij}}{r_{0}} \right) ^{m}}} \end{aligned}$$

where $r_{ij}$ and $r_{0}$ are the distance from atom *i* to atom *j* and its corresponding bond cutoff for the specific pair of atoms used to evaluate $r_{ij}$ with the strength parameters *n* and *m*, which is usually chosen based on the typical type of bond, in this work set to be 6 and 12 which were adopted from previous work [70]. The values for $r_{0}$ were set to be 2.65 ${\text{\AA} }$ for the C-C bond and 2.22 ${\text{\AA} }$ for other atomic interactions determined from distribution functions [70].

**Fig. 4:**
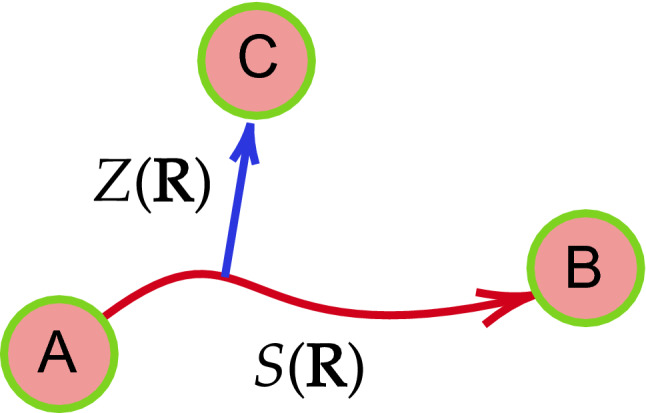
Path $S(\mathbf{R} )$ connecting reactant state A and product state B, and distance $Z(\mathbf{R} )$ from the reference path $S(\mathbf{R} )$ to metastable state C

The path-CV developed by Branduardi et al. [71] is another possible choice for a CV. The path-CV has been used to study complex chemical systems and calculate their associated FESs [72–74], allowing one to compute the progress along the high-dimensional reaction path $\varvec{R}$ and the distance $Z(\mathbf{R} )$ from the (high-dimensional) reference path (see Fig. 4). We refer the interested readers to Ref. [75] for more details.

In this work, matrices of reactant state A and product state B are used as initial and final frames in path-CV to generate the reference path $\varvec{R}$ for describing the evolution of the azaphilic addition. Contact matrices—adjacency matrices in which two atoms are adjacent if they are bonded—used for constructing path-CV of the iEDDA and azaphilic addition reactions are provided in the SI.

### Free Energy Surface Calculations

#### Metadynamics Simulation

WT-MetaD offers ways to use different and multiple CVs to calculate the FES of a given reaction. We adopt the WT-MetaD technique to treat iEDDA and azaphilic addition reactions performing WT-MetaD simulations with a number of possible combinations of the WT-MetaD parameters related to the Gaussian hills bias potential. For example, we varied the width and the height of the potential that the WT-MetaD employs to force the system away from the trap and travel to unexplored regions in the phase space. Table S1 in the SI shows a list of the potential parameters used, including deposited Gaussian potential, frequency or rate of hill deposition (pace), and upper and lower walls of the CV which are values that limit the region of the phase space (a function of CV) accessible during the sampling. In addition, we have checked whether the upper or lower walls have influenced our WT-MetaD calculations, and all the free energy landscapes calculated in our work are not affected by the upper or lower walls employed, providing therefore reliable reaction pathways and related energies.

**Fig. 5:**
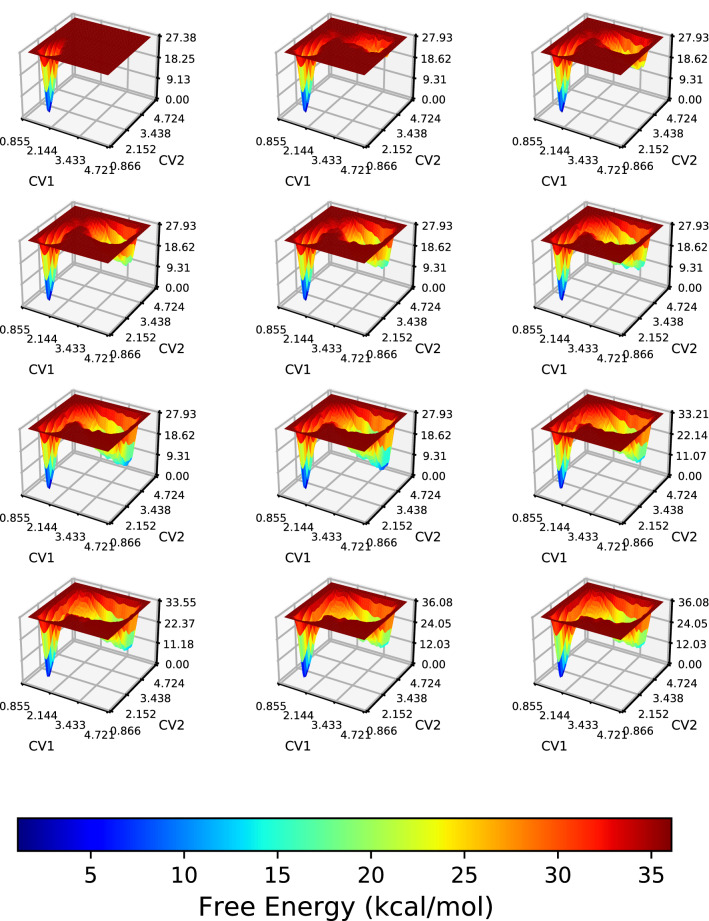
Time-progress FES of the first occurrence of iEDDA reaction in DCM solution for **Ph** derivative using WT-MetaD and C–C bond distances (CV1 and CV2) as CVs. The phase space starts with the product state and proceeds to the reactant state. Frame order: from left to right, top to bottom. The time difference between plots is 0.5 ps

*iEDDA Reaction* An important visualization of the evolution of the enhanced sampling simulation of the iEDDA reaction for the **Ph** derivative can be seen in Fig. 5. The time-progress profile of the FES shows a continuous biased energy surface and points out the existence of the product and reactant, respectively, as the former was the starting state. As can be seen from the first few snapshots, the system was activated by filling potentials and the process continued to the reactant after the simulation has been done for 2 ps. After that, the obtained FES evolution proceeds until the phase space is stationary and two metastable states were found within a defined region restricted by the wall parameter in the WT-MetaD simulation setup. Moreover, convergence is achieved when the simulation freely moves between the states of interest. This is equivalent to the reoccurring observation of the chemical transformation of interest, showing multiple back-and-forth events which refer to the occurrence of the desired iEDDA reaction. We deliberately went beyond the simplification in this study in order to get a complete perspective of both the reactant and product states for the relevant reactions, as well as their free energy differences in comparison with experimental observes.

**Fig. 6:**
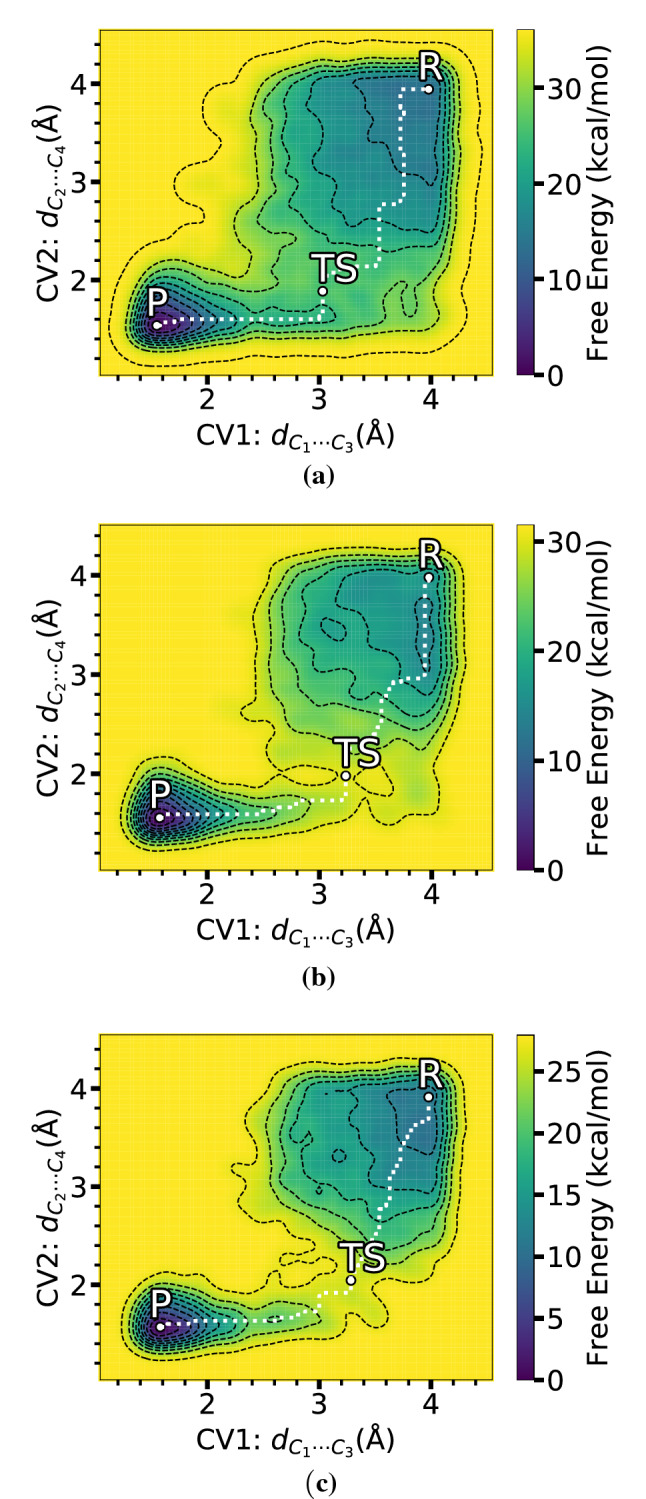
FESs of iEDDA reaction of **a** **Ph**, **b** **Me**, and **c** **H** derivatives using WT-MetaD and the two bond distances as CVs. A white dash line indicates the MEP passing through local minima and TS

**Fig. 7:**
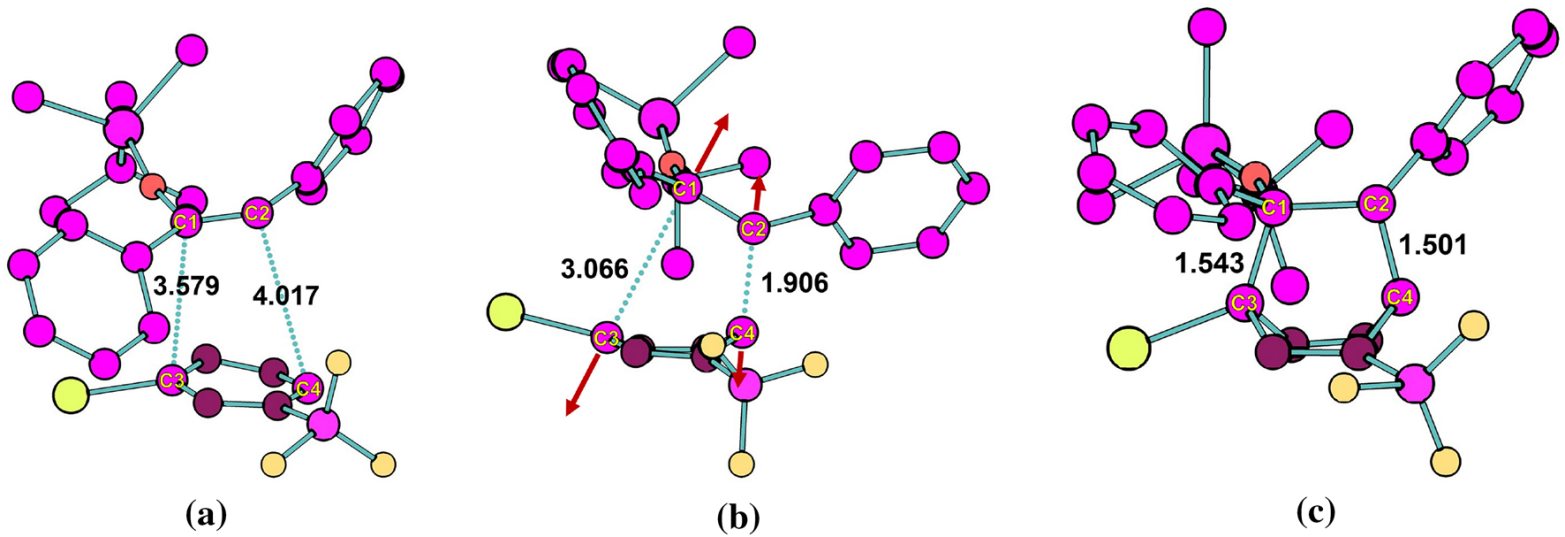
Selected reactant, TS, and product structures of iEDDA addition for **Ph** derivative in DCM solution obtained by metadynamics using the two bond distances as CVs. Bond distances are in ${\text{\AA} }$. The red arrows denote the vibrational motion observed around the TS. The snapshots were generated from the production phase of converged WT-MetaD simulation

The energy landscape using $\hbox {C}_1-\hbox {C}_3$ and $\hbox {C}_2-\hbox {C}_4$ distances as CVs in Fig. 6 clearly shows two separate local minima of reactant and product states of the [4+2] iEDDA reaction on 3-Br-*s*-tetrazine, and minimum energy path (MEP) calculated using MEPS [76]. Structures of reactant, TS, and product corresponding to states of the FES taken from the WT-MetaD trajectory are shown in Fig. 7. The activation energies (free energy barrier between reactant and TS) obtained from the first occurrence of the reaction from WT-MetaD using bond distances as CVs for **Ph**, **Me**, and **H** derivatives (without $\hbox {BF}_{3}$) are about 26.72 kcal/mol, 25.83 kcal/mol, and 18.76 kcal/mol, respectively. In addition, SPRINT and path-CV give the activation energies of the iEDDA reaction for **Ph** derivative of 27.22 kcal/mol and 33.36 kcal/mol, respectively, clearly showing that both of these CVs suggest that the iEDDA reaction is unfavorable when the phenyl is used as a substituent. However, when $\hbox {BF}_{3}$ adduct is used, the SPRINT coordinates indicate a contrary preference of azaphilic addition (20.02 kcal/mol) over the iEDDA reaction (24.94 kcal/mol) for **Ph** derivative in agreement with experimental findings [4]. The energetic preferences of azaphilic addition and iEDDA reactions (however without $\hbox {BF}_{3}$) using SPRINT coordinates are as follows: 28.28 vs 24.28 kcal/mol for **Me** and 22.80 vs 22.01 kcal/mol for **H**. Those reported values are in very good agreement with the values obtained by means of static DFT calculations (see Table 2). We note that the $\hbox {BF}_{3}$ adducts of **Me** and **H** derivatives were not considered in this work, which hampers direct comparison to experiment, but we refer the reader to Ref. [4] for more details on experimental observation and associated static DFT calculations. Another remark also shows that the SPRINT coordinates can estimate free energies of the iEDDA reaction that are in good quantitative agreement with the DFT-2 setting for static DFT calculation.

**Table 2:** Comparison of the free energy changes of reactant to TS ($E_{R-TS}$) of iEDDA reaction. The Gibbs $E_{R-TS}$ were computed by static DFT (in gas phase) while the Helmholtz $E_{R-TS}$ were obtained from WT-MetaD and BM calculations. The corresponding DFT energies for the geometry optimized structures using COSMO model are provided in parenthesis. DFT-1 denotes B3LYP/def2-TZVP//BP86/def2-TZVP settings, whereas DFT-2 denotes PBE/def2-SVP. Free energies are in kcal/mol

Dienophile	Adduct	DFT-1	DFT-2	WT-MetaD			BM
				Distance	SPRINT	path-CV	
Ph	–	29(27)	25(24)	26.72	27.22	33.36	20.48
Ph	N-1	23(20)	21(19)	24.05	23.59	–	–
Ph	N-2	23(20)	21(19)	24.33	24.94	–	–
Ph	N-4	21(20)	20(17)	20.26	28.25	–	–
Ph	N-5	30(28)	25(23)	27.21	29.44	–	–
Me	–	26(23)	22(20)	25.83	24.28	26.58	17.18
H	–	18(15)	17(15)	18.76	22.01	21.60	12.57

**Fig. 8:**
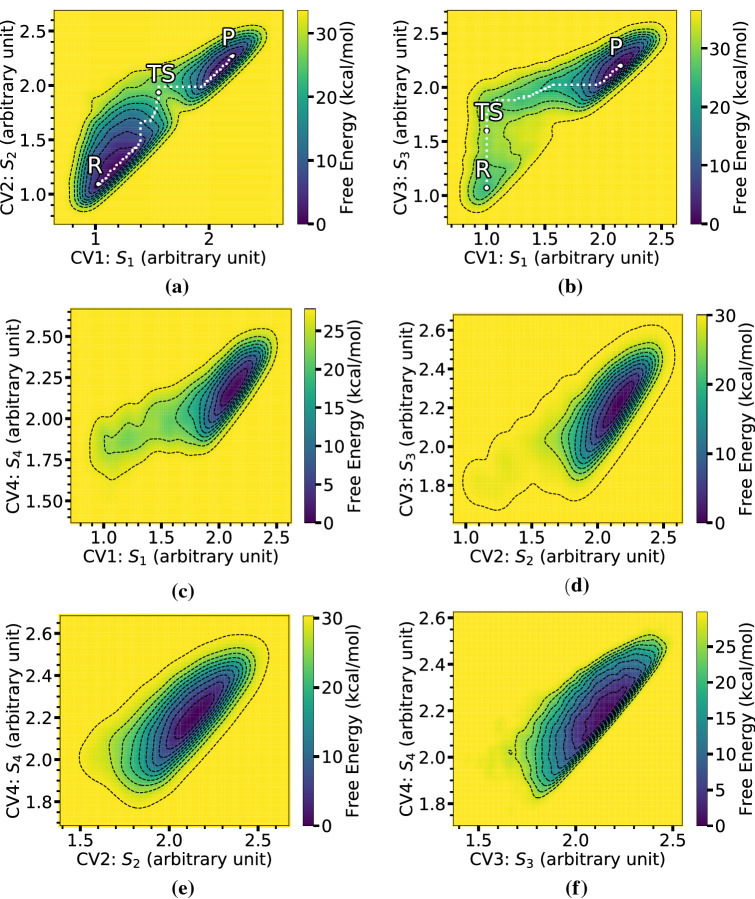
FESs of iEDDA reaction for **Ph** derivative in DCM solution obtained using WT-MetaD and different SPRINT coordinate sets as CVs: **a** $S_{1}$ and $S_{2}$, **b** $S_{1}$ and $S_{3}$, **c** $S_{1}$ and $S_{4}$, **d** $S_{2}$ and $S_{3}$, **e** $S_{2}$ and $S_{4}$, and **f** $S_{3}$ and $S_{4}$. A white dash line indicates the MEP passing through local minima and TS

Figure 8 shows FESs as a function of different SPRINT coordinates chosen from four carbon atoms (in both diene and dienophile) involving bond breaking in the iEDDA reaction. A list of pairs of the SPRINT coordinates is shown in Table 3. Each pair is selected based on the importance of the SPRINT coordinate that is defined as the actual value of SPRINT sorted from the highest coordinate ($S_1$) to the lowest coordinate ($S_4$). According to the property of SPRINT, each SPRINT coordinate can carry global information of the topology of the network and includes local information of surrounding atoms to the SPRINT coordinate of interest. As shown in the figure, only the first two FESs using $S_1$/$S_2$ (see 8a) and $S_1$/$S_3$ (see 8b) can reasonably reconstruct the energy pathway for iEDDA from product to reaction (reversely), especially, in the case of $S_1$/$S_2$ the metastable states can be distinguished. On the other hand, the reconstruction ability of WT-MetaD to reach the desired FES decreases when the low-value SPRINT coordinates, such as $S_2$/$S_4$ (see 8e) and $S_3$/$S_4$ (see 8f), are used as biased CVs, losing the possibility to find local minima of the state of interest. This also shows the impact of reordering the SPRINT coordinates that are associated with different atoms. It is worth mentioning that in the cases where the simulation is unable to quantify the desired metastable states and when the free energy difference between the reactant and product states is not of interest, it is common to stop MetaD simulations after an unexpected reaction is found.

**Table 3:** Pairs of selected SPRINT coordinates used as CVs for the iEDDA reaction. Note that the importance of SPRINT coordinates is sorted from $S_{1}$ to $S_{4}$ (for details, see text)

No. of Pair	SPRINT coordinates
1	$S_{1}$ & $S_{2}$
2	$S_{1}$ & $S_{3}$
3	$S_{1}$ & $S_{4}$
4	$S_{2}$ & $S_{3}$
5	$S_{2}$ & $S_{4}$
6	$S_{3}$ & $S_{4}$

A few comments can be added on the relationship between the evolution of the CVs and structural change of the TS of the iEDDA reaction. As can be seen in Fig. 6, all FESs obtained with C-C distances as CVs show that the iEDDA reactions using **Ph**, **Me**, and **H** derivatives share the same reaction pathway. As the simulation started in in the product state with stationary $C_{1}{\cdots }C_{3}$ and $C_{2}{\cdots }C_{4}$ distances of 1.60 ${\text{\AA} }$, the system was then forced to travel to the nearby region along the CV1 ($C_{1}{\cdots }C_{3}$) rather than CV2 ($C_{2}{\cdots }C_{4}$), from about 1.60 ${\text{\AA} }$ to 3.20 ${\text{\AA} }$ for CV1. The simulation then proceeded to the TS with a slight increase of CV2 from about 1.75 ${\text{\AA} }$ to 2.09 ${\text{\AA} }$. In contrast, for the route from the TS to reactant state, the system was instead mainly governed by the $C_{2}{\cdots }C_{4}$ distance with a drastic change of CV2 from 2.09 to 3.95 ${\text{\AA} }$ and of CV1 from 3.20 to 4.00 ${\text{\AA} }$. The overall evolution of the reaction from product to reactant states and vice versa corresponds to the oscillation of $C_{1}{\cdots }C_{3}$ and $C_{2}{\cdots }C_{4}$ bonds. When comparing with the FESs using $S_1$/$S_2$ and $S_1$/$S_3$ CVs, the pathways of the iEDDA reaction shown in Fig. 8a and b manifest the direct route via the TS which is located in a narrow region. Both of these two SPRINT coordinates’ FESs are symmetric and show identical characteristic of the stable states compared to other SPRINT coordinates’ FESs such as $S_1$/$S_4$ since the SPRINT coordinates start from around 0.8 to 2.6 (arbitrary unit) in both directions ($S_{1}$/$S_{2}$ and $S_{1}$/$S_{3}$). Another interesting difference between these two FESs is the location of the TS. In the case of $S_{1}$/$S_{2}$, the TS was found to be in between the reactant and product states, indicating the balance between the $S_{1}$ and $S_{2}$ coordinates that they are almost equal to each other in terms of the importance of the components in SPRINT coordinates, while in the other case where $S_{3}$ was used instead the TS was shifted to the lower $S_{1}$ and $S_{3}$ components, corresponding to the imbalance between $S_{1}$ and the lower-important component, i.e., $S_{3}$ or $S_{4}$.

**Fig. 9:**
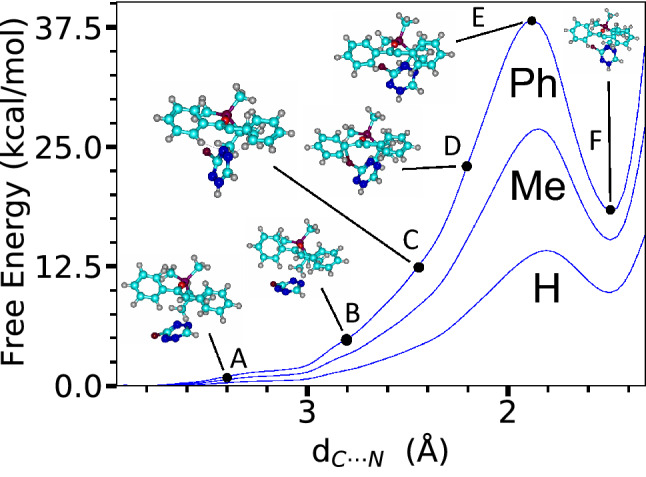
FES of the azaphilic addition in DCM solution (no $\hbox {BF}_{3}$ adduct) using WT-MetaD and C-N distance (**Ph**, **Me**, and **H**). C-N distances (${\text{\AA} }$) for each structure are as follows: **A** 3.40, **B** 2.81, **C** 2.42, **D** 2.22, **E** 1.90, and **F**: 1.50

**Fig. 10:**
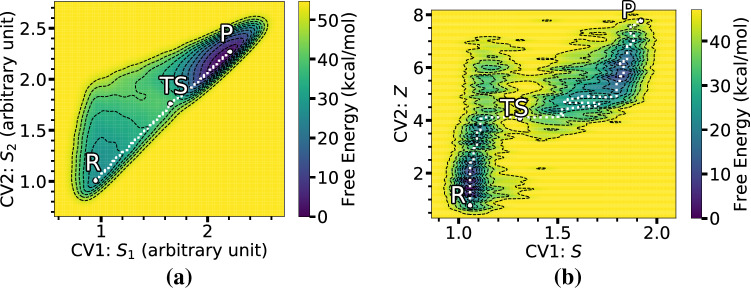
FESs of the azaphilic addition for **Ph** in DCM solution using WT-MetaD and different CVs: **a** SPRINT coordinates and **b** path-CV. A white dash line indicates the MEP passing through local minima and TS

**Fig. 11:**
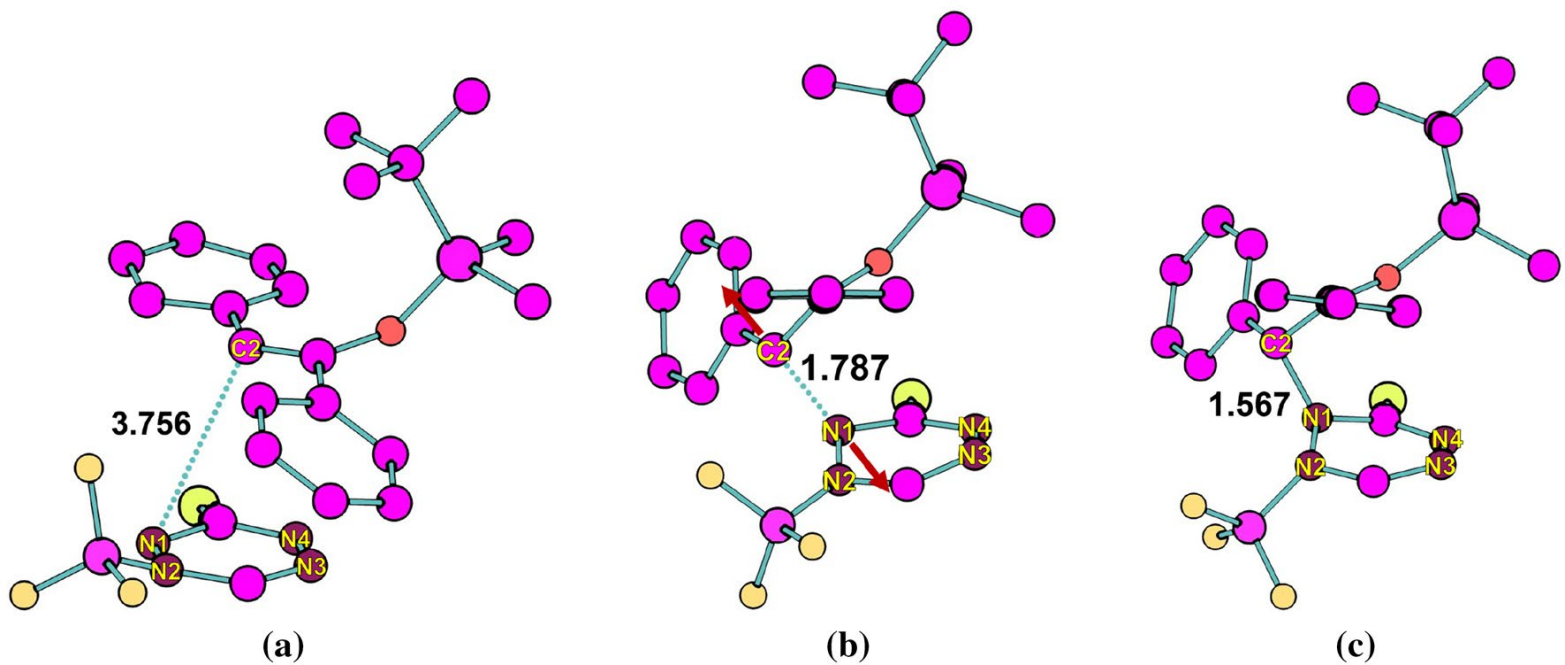
Selected reactant, TS, and product structures of azaphilic addition for **Ph** derivative in DCM solution obtained by metadynamics using the two bond distances as CVs. Bond distances are in ${\text{\AA} }$. The red arrows denote the vibrational motion observed around the TS. The snapshots were generated from the production phase of converged WT-MetaD simulation

*Azaphilic Addition Reaction* When **Me** substituted silyl-enol ether reacted with 3-Br-*s*-tetrazine, the pyridazine was observed as the minor product of this transformation in low conversion together with a major unknown compound [1]. When bulky partner **Ph** substituted silyl-enol ether has been used in the azaphilic addition, in this work, we found that the reaction changes from cycloaddition to azaphilic addition when using SPRINT coordinate as CVs and $\hbox {BF}_{3}$, mirroring the previous work which found that the latter is an energetically favored reaction rather than the former [4]. This is in line with the experimental finding in the previous work that the ability of the dienophiles for the azaphilic addition depends strongly on the steric demand of the alkyl and aryl substitutes [4].

The FESs of the azaphilic addition reaction obtained with the C-N distance as CV are shown in Fig. 9 with structures along the reaction pathway. The FESs obtained using SPRINT coordinates or path-CV as CVs are shown in Fig. 10. The MEP also shows the direct route from reactant to product states via the TS. In the particular case of the unexpected azaphilic addition reaction of the **Ph** derivative, the activation energies obtained from the FES using WT-MetaD simulation are about 37.62 and 32.79 kcal/mol for using the C-N bond distance and their SPRINT coordinates, respectively, as CVs. When the $\hbox {BF}_{3}$ catalyst was used, the activation energies for adducts with the $\hbox {BF}_{3}$ attached at N-1 and N-2 positions of the ring gradually decrease to 28.90 and 20.58 kcal/mol, respectively, for bond distance CV, and to 25.37 and 20.02 kcal/mol, respectively, for the SPRINT CVs. These activation energies are consistent with the static DFT’s energy barriers measured as free energy differences between the optimized geometry of the associated reactant and the TS. However, we also noticed that the dynamic simulations using the SPRINT CVs overall yield lower activation energies than using bond distance CV or path-CV. The difference of this estimated activation energy between using the SPRINT and other CVs might be attributed to the slightly different TS structures obtained, and/or localization of selected atoms in a molecule, which could directly affect the lower eigenvalue of the corresponding eigenvector of the adjacency matrix used to calculate the SPRINT. We also notice that path-CV gives the activation energy of about 24.62 kcal/mol for the **H** derivative azaphilic addition, whereas, the C-N distance gives the lowest free energy of 13.95 kcal/mol among other methods in Table 4. A large difference between the activation energies using the C-N distance and the path-CV (10.67 kcal/mol at most in the case of **H** derivative) indicates that the transformation of CV from internal coordinate to geometric coordinate could manufacture different characteristics of the FES of the same reaction.

**Table 4:** Comparison of the free energy changes of reactant to TS ($E_{R-TS}$) of azaphilic addition reaction. The Gibbs $E_{R-TS}$ were computed by static DFT (in gas phase) while the Helmholtz $E_{R-TS}$ were obtained from WT-MetaD and BM calculations. The corresponding DFT energies for the geometry optimized structures using COSMO model are provided in parenthesis. DFT-1 denotes B3LYP/def2-TZVP//BP86/def2-TZVP settings, whereas DFT-2 denotes PBE/def2-SVP. Free energies are in kcal/mol

Dienophile	Adduct	DFT-1	DFT-2	WT-MetaD			BM
				Distance	SPRINT	path-CV	
Ph	–	34(32)	32(30)	37.62	32.79	32.40	28.26
Ph	N-1	26(23)	27(24)	28.90	25.37	–	–
Ph	N-2	17(14)	23(21)	20.58	20.02	–	–
Me	–	27(23)	26(24)	25.14	28.28	28.92	21.65
H	–	24(22)	21(18)	13.95	22.80	24.62	16.53

In our previous study on the investigation of the electrophilicity of each backbone atom in 3-Br-*s*-tetrazine for the same kind of reaction, we have found that the abovementioned steric effect helps to rationalize the reactivity of 3-Br-*s*-tetrazine towards azaphilic addition against iEDDA reaction [4]. The most electrophilic site was identified as a nitrogen atom that is spatially opposite to the $\hbox {BF}_{3}$ attachment and found to undergo the azaphilic addition reaction. Moreover, the results revealed that nucleophilic attack on aromatic compounds without elimination is not favored, as aromaticity is not restored. Moreover, committor analysis was performed to describe the ability of bond distance as CVs in WT-MetaD simulations (see the SI for more details). The analysis clearly showed that the bond distances are reasonable to capture the transition between reactant and product for the iEDDA and azaphilic addition reactions.

*Structural Analysis* To gain additional insight into the evolution of both iEDDA and azaphilic addition reactions, further analysis is desirable for providing a quantitative estimation of the reaction progress. Root mean square deviation (RMSD) plots are general tools that can be used to track the structural change of molecules in the simulation and to measure the quality of reproduction of the reaction. RMSD values depend on the interaction between reaction partners. We computed the RMSD of atomic positions of selected studied derivatives using the trajectory from the biasing simulation (see the SI for all RMSD plots). Generally, a low value of individual RMSD reflects the high significant pose of the structure of interest onto the reference structure. Selected snapshots for nuclear structures of the reactant, TS, and product states obtained from the first occurrence of the reaction in WT-MetaD trajectory were used as the reference structures (see Figs. 7 and 11).

**Fig. 12:**
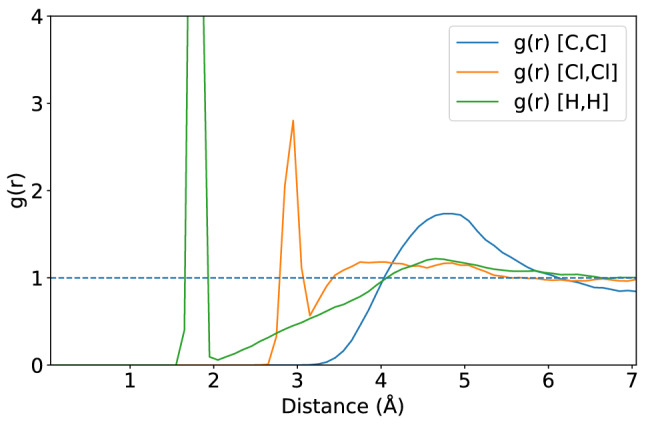
Partial *g*(*r*) of DCM solution with **Ph** derivative in the reactant state for C–C, Cl–Cl and H–H distances at 300 K and 1 Bar under PBC. The *g*(*r*) was computed with a spherical shell of thickness $\delta r$ of 0.01 ${\text{\AA} }$

**Fig. 13:**
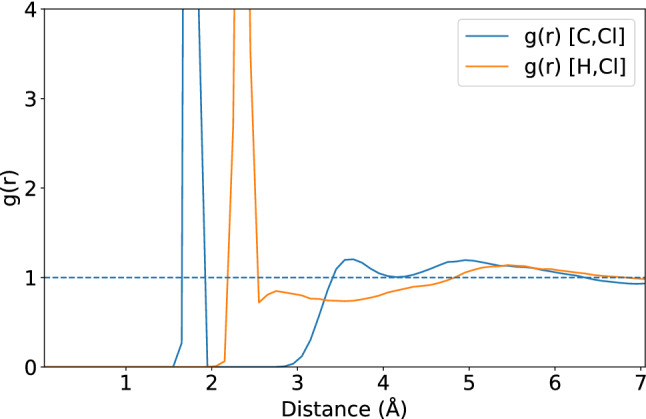
Partial *g*(*r*) of DCM solution with **Ph** derivative in the reactant state for C–Cl and H–Cl distances at 300 K and 1 Bar under PBC. The *g*(*r*) was computed with a spherical shell of thickness $\delta r$ of 0.01 ${\text{\AA} }$

Moreover, we computed the radial distribution functions *g*(*r*) for the **Ph** derivative to investigate the influence of the DCM solvent. In principle, the first peak of the computed *g*(*r*) represents the probability to find DCM in the corresponding first solvent layer (see Figs. 12 and 13). At the short distance from the reference point, the bulk DCM is calculated to be at around 1.5 ${\text{\AA} }$ to 3.0 ${\text{\AA} }$. We also found that the computed *g*(*r*) of DCM is closely related to the RMSD of the solute using the product state as the reference structure for both the iEDDA and azaphilic addition reactions at the initial biasing simulation for 1.9 ps. That is, the solute and DCM molecules were consolidated and remained in the structure of the product state which is the most stable state compared to other states in the trajectory. Moreover, during the course of the enhanced sampling simulation, DCM molecules undergo structural reorganization which then affects the stability of the structure of the solutes as we found a gradual increase of the rate of its RMSD movement (see the SI for more details). This finding is a piece of evidence that the explicit solvent in the enhanced sampling simulations plays a non-negligible role for the iEDDA and azaphilic addition reactions, which is not necessarily captured well in the static calculation where the implicit solvent continuum model was used.

#### Blue Moon Ensemble Simulation

**Fig. 14:**
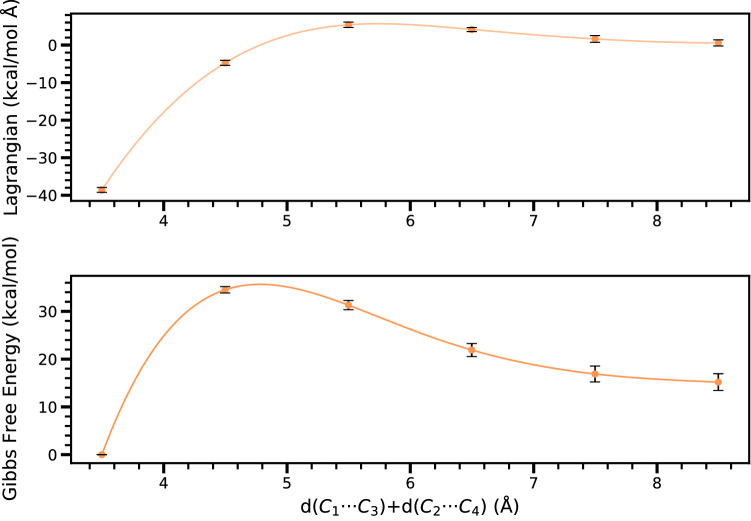
Free energy pathway of the iEDDA reaction of 3-Br-*s*-tetrazine for **Ph** derivative in DCM solution using BM ensemble simulation

In addition to the WT-MetaD method, the energy path quantified by the BM ensemble can provide the energy profile of the reaction. The BM ensemble is run in a similar way to that of the MetaD. We estimate the Lagrangian of the system at different constraints. Since all BM simulations of **Ph**, **Me**, and **H** share the computational workflow and analysis, the case of **Ph** is selected as representative for results and discussion. The Lagrangian (constraint forces) and free energy obtained from the BM simulations for **Ph** as a function of bond length are shown in Fig. 14 for $d(C_{1} \cdots C_{3}) + d(C_{2} \cdots C_{4})$. The structure of reactant, TS, and product corresponding to the local minima and maxima are consistent with those obtained by the MetaD, confirming the consistency in terms of phase space. The corresponding activation energies by means of BM ensemble for iEDDA and azaphilic additions of **Ph**, **Me**, and **H** are included in Tables 2 and 4, respectively.

A direct comparison of activation energies clearly indicates the feasibility of the two different reaction pathways in good agreement with static DFT calculation and the WT-MetaD simulations in terms of qualitative tendency, in ascending order of the size of substituent: H, to Me, and Ph. The overall free energies of the iEDDA reaction of substrates without $\hbox {BF}_{3}$ decrease from 20.48 to 17.18, and to 12.57 kcal/mol, respectively. When the iEDDA reaction takes place in the presence of the $\hbox {BF}_{3}$ catalyst, the energy barrier is reduced. However, in the case of azaphilic addition, all free energies are still high compared to the iEDDA reaction. For example, BM simulation of azaphilic addition using H as substituent gives free energy of 16.53 kcal/mol, which is close to that of 17.18 kcal/mol for the iEDDA reaction using Me as substituent. In contrast to WT-MetaD simulations, the BM method predicts in most cases the activation energies less quantitatively accurate compared to the values obtained by all other CVs utilized in the WT-MetaD, showing the weakness of this constraint-based ensemble method [63]. In addition, underestimation of the energy barrier on the FES computed by BM ensemble is noted which might be due to the fact that the bond distance used as constraint does not capture the exact transition state (TS) structure. Therefore, the qualitative accuracy in the calculation of the free energy by BM ensemble depends on the resolution and the degree of freedom of the constraint used in the simulation.

## Conclusion

In this work, we computationally investigate the chemical reactivity of inverse electron demand Diels–Alder (iEDDA) and nucleophilic (azaphilic) addition reactions between 3-Br-*s*-tetrazine (with or without $\hbox {BF}_{3}$ adduct) and alkyl substituted silyl-enol ethers in dichloromethane (DCM) solvent. The effect of Phenyl (**Ph**), Methyl (**Me**), and Hydrogen (**H**) substituents on dienophile is also systematically investigated. Enhanced sampling-based metadynamics (MetaD) and blue moon (BM) ensemble were used to reconstruct free energy surfaces (FESs) and calculate activation energies. The dynamic calculations can provide a comprehensive perspective of the studied reaction in terms of FESs with the description of explicit DCM solvent at ambient conditions, whereas in static DFT calculation explicit solvent can cause unreliability of the estimation of activation energy [77, 78].

We adopted the Well-Tempered MetaD (WT-MetaD) that can help where standard MetaD lacks the convergence in the reconstruction of FESs in a single simulation run. The convergence of the simulation is analyzed in order to characterize the multidimensional free energy landscape of the system and to understand rare events. A direct comparison between energetic differences of the reaction pathway simulated by either WT-MetaD method, BM ensemble, or static DFT model is mirroring in terms of quantitative accuracy with respect to the different substituents (**Ph**, **Me**, and **H**) and the trend of reaction feasibility. There is a remarkable agreement among the nuclear structures of the reactant, transition state, and product. We also found that the steric hindrance in the ligand of dienophile plays an important role in the selectivity of the reaction.

In comparison to previous static DFT calculations with implicit DCM solvent, structural analysis on the configuration of explicit DCM molecules in WT-MetaD shows that dynamic reorganization of DCM can stabilize the interaction between solute and solvent. The RMSDs with different reference states were also computed to study the transformation of the iEDDA and azaphilic molecules via the reaction pathway. The crossing of RMSDs between reactant and product references shows that the reaction does not take place immediately but is delayed after (small) rearrangement of DCM solvent molecules [79].

There is a promising chance to explore the ambiguous chemistry of *s*-tetrazines and silyl-enol ethers using other collective variables (CVs) even though those were developed for specific problems. We gain a much better understanding of the importance of structural descriptors from dynamic simulations, especially helping one carefully choosing the appropriate CVs for similar DA and azaphilic addition reactions or other related chemical reactions. Our computational WT-MetaD results reveal that internal-based CVs used in this work, SPRINT coordinate and path-CV, yield free activation energies which are in good agreement with energies obtained using bond distance as CV. We believe that the current study will pave the way for further either short-term or long-term studies, leading to the novel discovery of and expanding the understanding of both iEDDA and azaphilic addition reactions.

## Supplementary Information

Below is the link to the electronic supplementary material.

Supplementary file1 (PDF 3024 kb)
