# Supplementary material for: Closer Look at Inverse Electron Demand Diels–Alder and Nucleophilic Addition Reactions on s-Tetrazines Using Enhanced Sampling Methods
Source: Top Catal. 2021 Oct 23;65(1-4):1–17. doi: 10.1007/s11244-021-01516-y (PMC8816378; doi:10.1007/s11244-021-01516-y)
Supplement: Supplementary file 1 — Supplementary file1 (PDF 3024 kb) [file 11244_2021_1516_MOESM1_ESM.pdf]

Supporting Information: Closer Look At Inverse Electron Demand Diels-Alder and Nucleophilic Additions on s-Tetrazines Using Enhanced Sampling Methods

Rangsiman Ketkaew, Fabrizio Creazzo, and Sandra Luber*

Department of Chemistry, University of Zurich, Winterthurerstrasse 190, CH-8057 Zürich, Switzerland

E-mail: sandra.luber@chem.uzh.ch

1 Simulation box

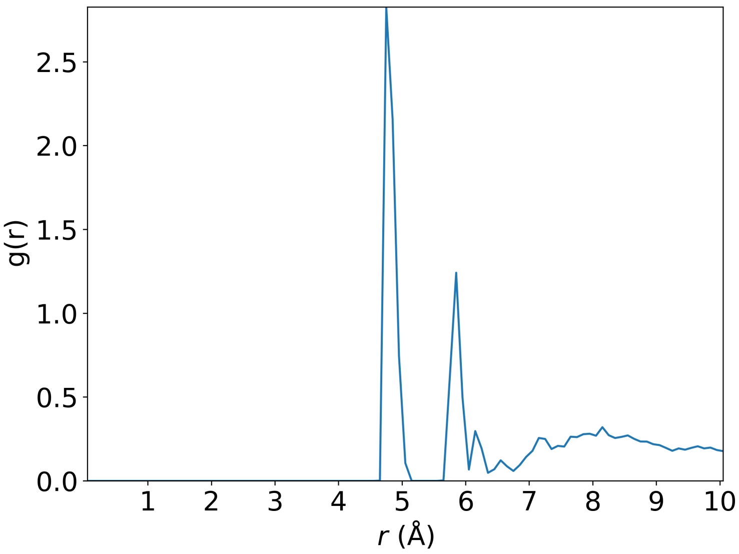

Figure S1: Radial distribution function of C-C of pure dicholoromethane simulation system using GFN-xTB MD simulation.

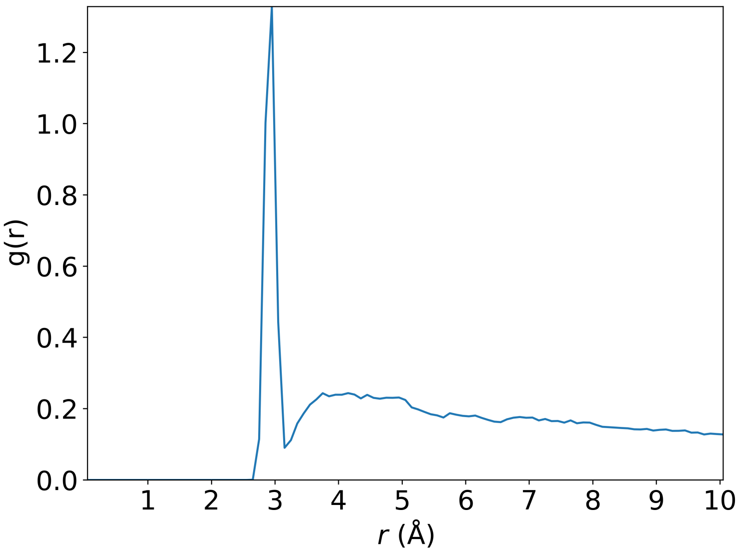

Figure S2: Radial distribution function of Cl-Cl of pure dicholoromethane simulation system using GFN-xTB MD simulation.

2 Properties calculations

2.1 The change of enthalpy of vaporization

The change of enthalpy of vaporization (ΔH_{vap}) of the simulation system from the first law of thermodynamics[S1] as follows:

$$U_{vap} = \ U_{gas} - U_{liq} \tag{1}$$

$$H_{vap} = \ \ < U_{gas} > - < U_{liq} > + nRT \tag{2}$$

$$\Delta H_{vap} = \ E_{gas} - \frac{E_{liq}}{N} + k_B T \tag{3}$$

where U is internal energy, H is enthalpy, E is potential energy, R is the gas constant (8.3145 $J{\cdot}mol^{-1}{\cdot}K^{-1}$), T is the thermodynamic temperature of the system, k_B is the Boltzmann constant, and $< \cdots >$ indicates average over the number of particles (N) in the system.

2.2 Viscosity

Viscosity η is calculated from the diffusion coefficient:

$$D = D_0 - \frac{k_b T \xi}{6\pi\eta L} \tag{4}$$

where D and D_0 are diffusion coefficients at simulation time t and at initial time t_0, respectively. Other parameters are: the absolute temperature T (in Kelvin) of the system, simulation box length L, and fitting parameter ξ. We note that $\xi \approx 2.837297$ reported in Ref. S2 is used in this study. D was derived from Einstein's random walk model that

correlates the diffusion to the mean squared displacement (MSD) of atomic position[S3] as follows:

$$\lim_{t \to \infty} MSD(t) = 6Dt \tag{5}$$

where t is simulation time.

3 Metadynamics

3.1 Biasing parameters

Table S1: Gaussian potential parameters: width (σ) and height (w) used to enhance sample the metastable state in a given region with a boundary defined by upper and lower walls (in arbitrary units), at a constant temperature of around 300 K and adopting a bias factor of 25 for well-tempered metadynamics. Rate of hill addition (pace) is also provided.

CV	Height (kJ)	Width (Å)	Pace	Upper wall	Lower wall
d_1 & d_2	2	0.05 & 0.05	50	4.5	1.0
d_1 & d_2	4	0.05 & 0.05	50	4.5	1.0
d_1 & d_2	4	0.05 & 0.05	100	4.5	1.0
S_1 & S_2	4	0.01 & 0.01	50	3.0	1.0
S_1 & S_2	4	0.01 & 0.01	100	3.0	1.0
S_1 & S_2	4	0.05 & 0.05	50	3.0	1.0
S_1 & S_2	4	0.05 & 0.05	100	3.0	1.0
S_1 & S_2	2	0.05 & 0.05	50	3.0	1.0
S_1 & S_2	2	0.05 & 0.05	100	3.0	1.0
S_1 & S_3	2	0.05 & 0.05	50	3.0	1.0
S_1 & S_3	2	0.05 & 0.05	50	3.0	1.0
S_2 & S_3	2	0.05 & 0.05	50	3.0	1.0
S_2 & S_4	2	0.05 & 0.05	50	3.0	1.0
S_3 & S_4	2	0.05 & 0.05	50	3.0	1.0
path-CV	5	0.05 & 0.05	50	5	-
path-CV	10	0.05 & 0.05	100	5	-
path-CV	10	0.05 & 0.15	100	5	-

3.2 Construction of path-CV

- Contact matrix for iEDDA reaction.

```
ROWS 4 COLS 8 FRAMES 2
ROWSLABEL C1 C2 C3 C4
COLSLABEL C H B N O F Si Br
FRAME 1
```

```
    2.0  0.0  0.0  0.0  1.0  0.0  0.0  0.0
    2.0  1.0  0.0  0.0  0.0  0.0  0.0  0.0
    0.0  0.0  0.0  2.0  0.0  0.0  0.0  1.0
    0.0  1.0  0.0  2.0  0.0  0.0  0.0  0.0
FRAME 2
    3.0  0.0  0.0  0.0  1.0  0.0  0.0  0.0
    3.0  1.0  0.0  0.0  0.0  0.0  0.0  0.0
    1.0  0.0  0.0  2.0  0.0  0.0  0.0  1.0
    1.0  1.0  0.0  2.0  0.0  0.0  0.0  0.0
```

- Contact matrix for azaphilic attack.

```
ROWS 4 COLS 8 FRAMES 2
ROWSLABEL C1 C2 C3 C4
COLSLABEL C H B N O F Si Br
FRAME 1
    2.0  0.0  0.0  0.0  1.0  0.0  0.0  0.0
    2.0  1.0  0.0  0.0  0.0  0.0  0.0  0.0
    0.0  0.0  0.0  2.0  0.0  0.0  0.0  1.0
    0.0  1.0  0.0  2.0  0.0  0.0  0.0  0.0
FRAME 2
    2.0  0.0  0.0  0.0  1.0  0.0  0.0  0.0
    2.0  1.0  0.0  1.0  0.0  0.0  0.0  0.0
    1.0  0.0  0.0  2.0  0.0  0.0  0.0  1.0
    1.0  1.0  0.0  2.0  0.0  0.0  0.0  0.0
```

4 Structural analysis

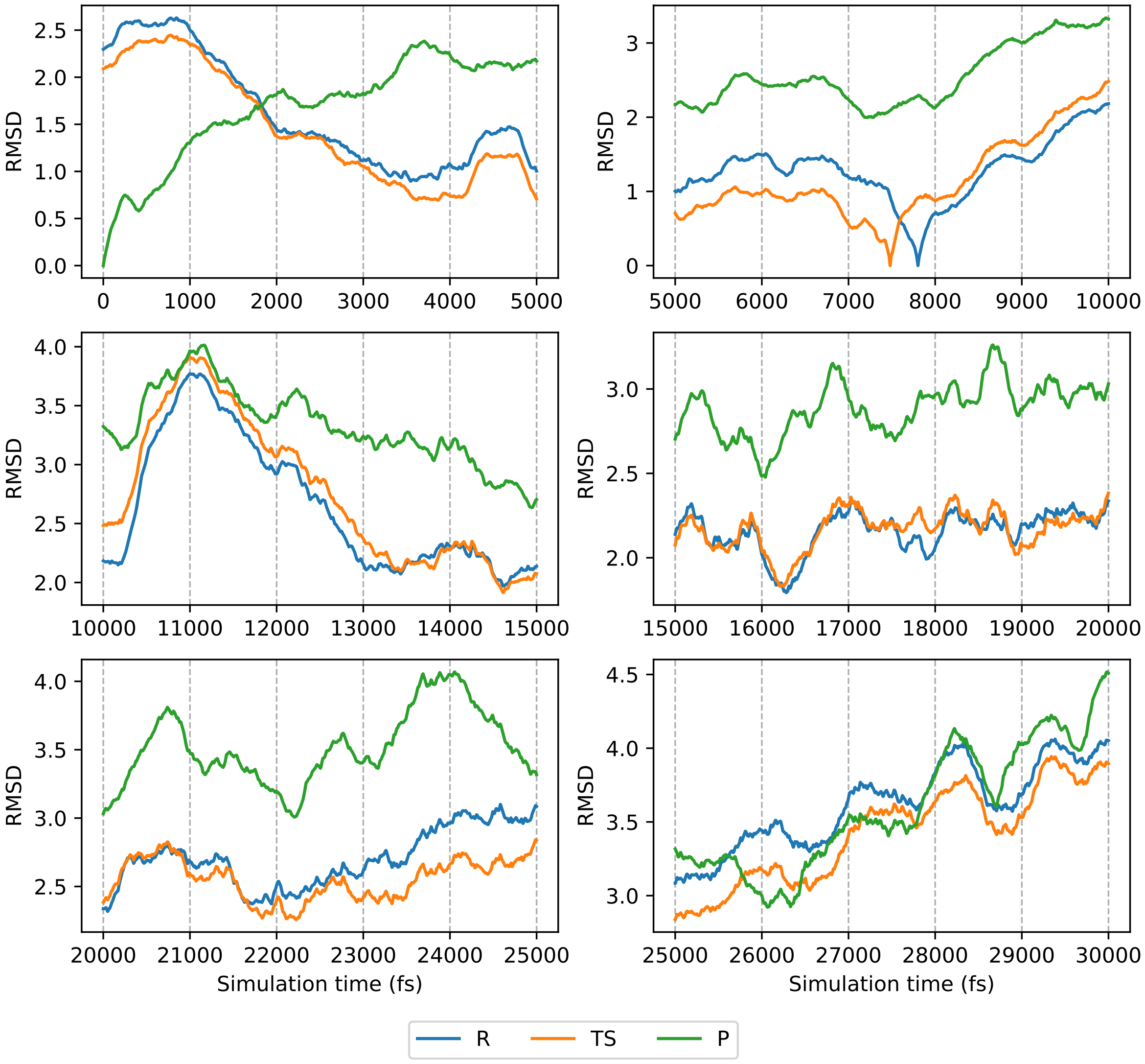

Figure S3: RMSD of atomic positions as a function of simulation time of **H** derivative for iEDDA reaction in DCM solution using WT-MetaD with $C_1 \cdots C_3$ and $C_2 \cdots C_4$ distances as CVs. Reactant (R), transition state (TS), and product (P) structures from the first occurrence of the reaction in the trajectory are used as reference structures.

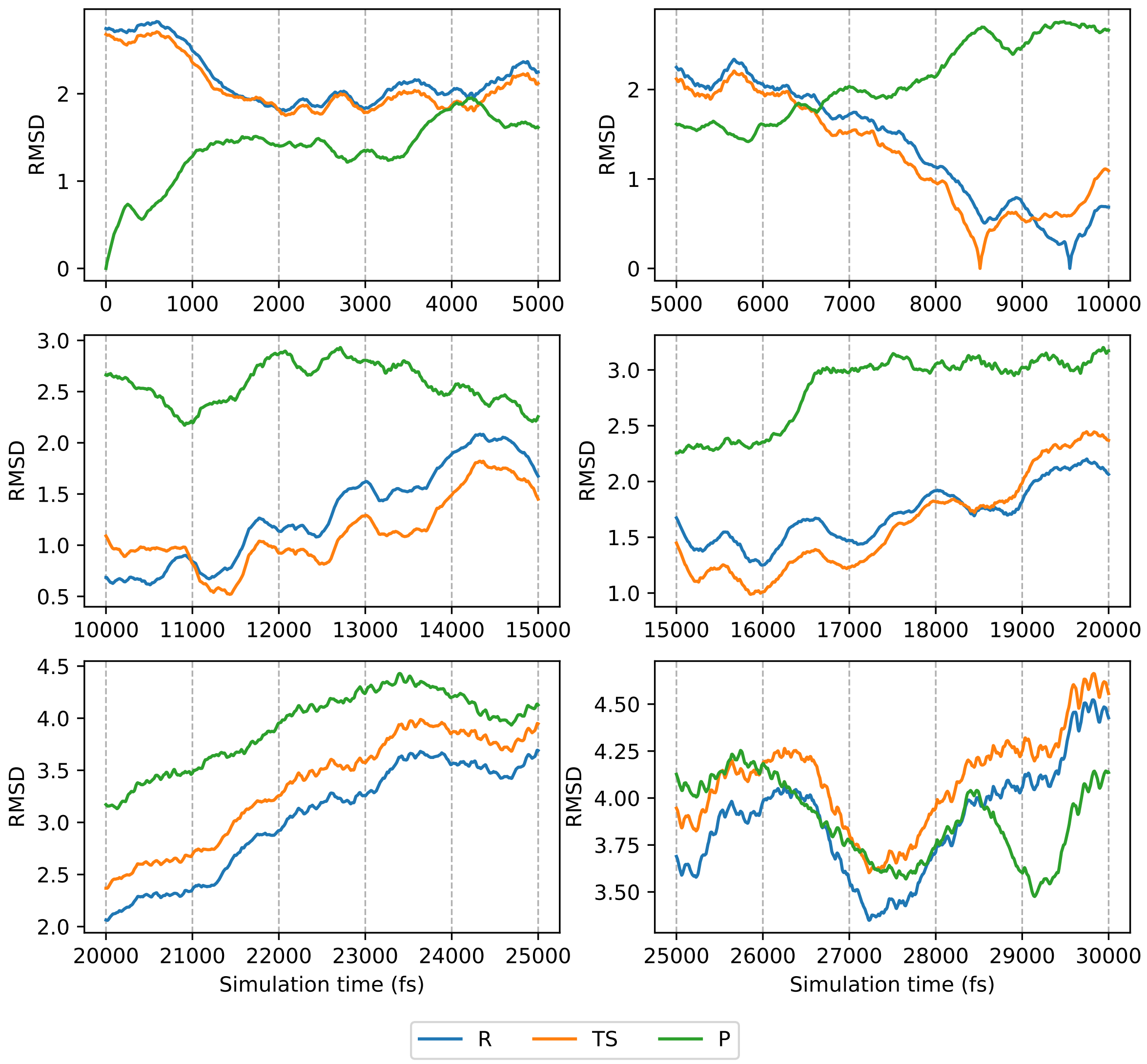

Figure S4: RMSD of atomic positions as a function of simulation time of **Ph** derivative for azaphilic attack in DCM solution using WT-MetaD with $C_2 \cdots N_4$ distance as CV. Reactant (R), transition state (TS), and product (P) structures from the first occurrence of the reaction in the trajectory are used as reference structures.

5 Convergence analysis

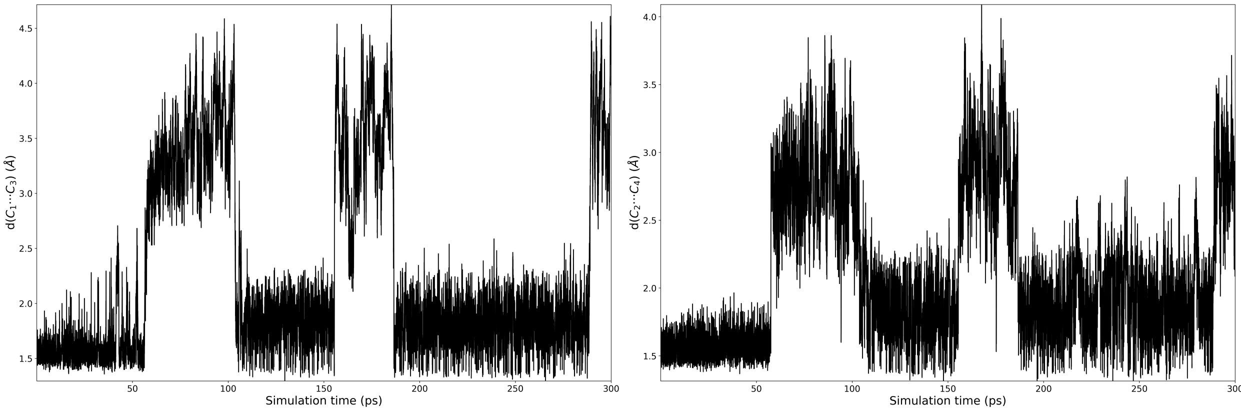

(a) (b)

Figure S5: (a) $C_1 \cdots C_3$ and (b) $C_2 \cdots C_4$ distance profiles of iEDDA reaction for Phenyl derivative in DCM solution using MetaD.

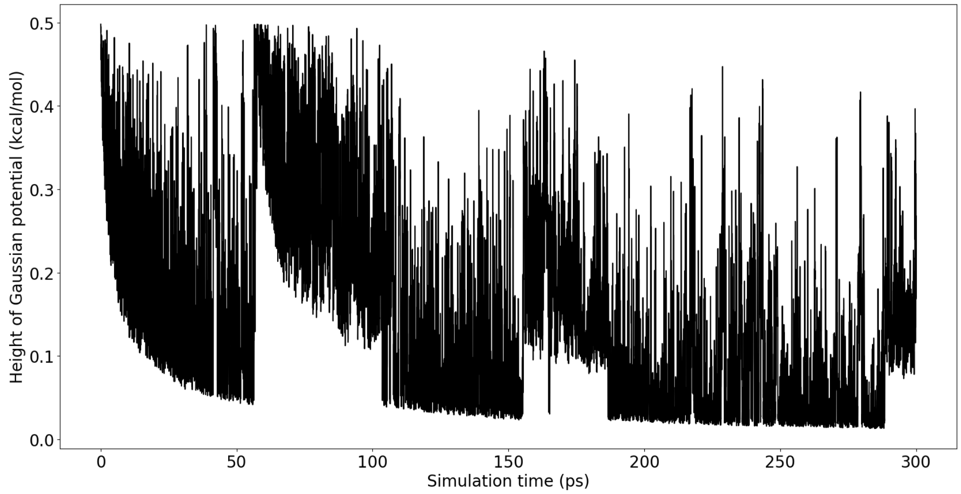

Figure S6: Height of the deposited Gaussian potential as a function of simulation time showing the tendency of the achievement of energy convergence of different local minima of iEDDA reaction on free energy surface.

In-depth analysis on the convergence of enhanced sampling is intensively required to understand the relation between the existence of metastable states and their corresponding statistics.[S4,S5] The convergence of MetaD and BM ensemble plays an important role in de-

ciding how much one can trust the outcome from the simulations. Convergence typically happens when the simulation freely moves from one metastable state to another, broadening the scope of molecular dynamics observations. This is analogous to the frequent discovery of the chemical transition state which can be assessed by static calculation. Moreover, if the free energy difference between the reactant and the sample is not of concern, MetaD simulations can also be stopped after the first reaction of interest has occurred. In this work, we actively obtain a description of both reactants with different substitution and their free energy gap, and we are going beyond this simplification.

Upon the existence of the abovementioned product species, a further indication of convergence of MetaD simulation related to studied chemical reactions is obtained by considering the progress of statistical dynamics of biased potentials used to drive the system. Figure S6 shows the change of height of Gaussian potential deposited along CVs coordinate as a function of simulation time of the WT-MetaD simulation. In the initial simulation time, the system remains in the first metastable state and then transforms to the second metastable state, corresponding to the drastic increase of C-C distances (between diene and dienophile) at around 50 ps of our metadynamics run. However, one needs to continue the simulations until all of the recursive patterns of the desired reaction have been found in order to confirm the according to convergence.

6 Committor analysis

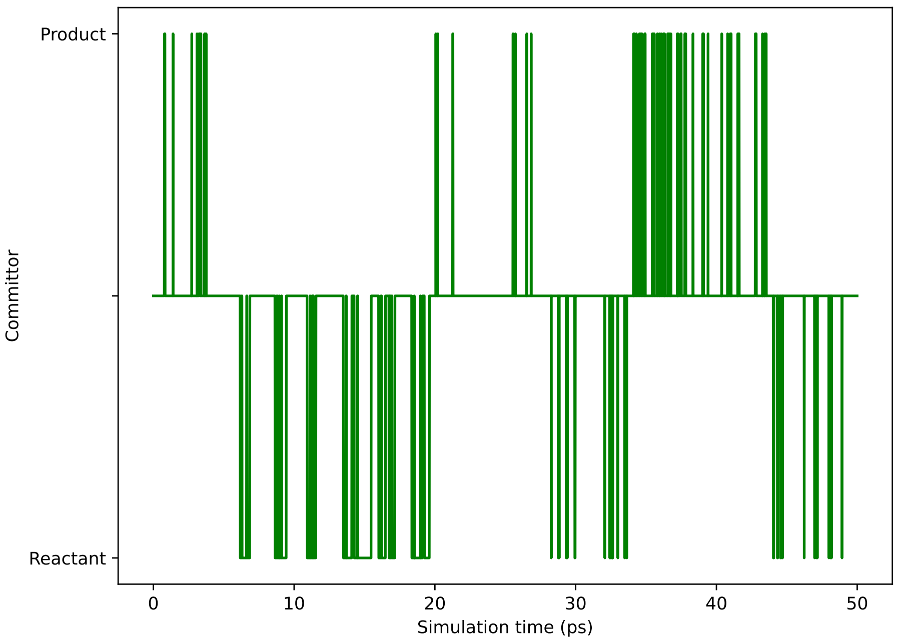

Figure S7: Committor profile for the iEDDA reaction for Phenyl derivative in DCM solution using MetaD bond distance as a CV.

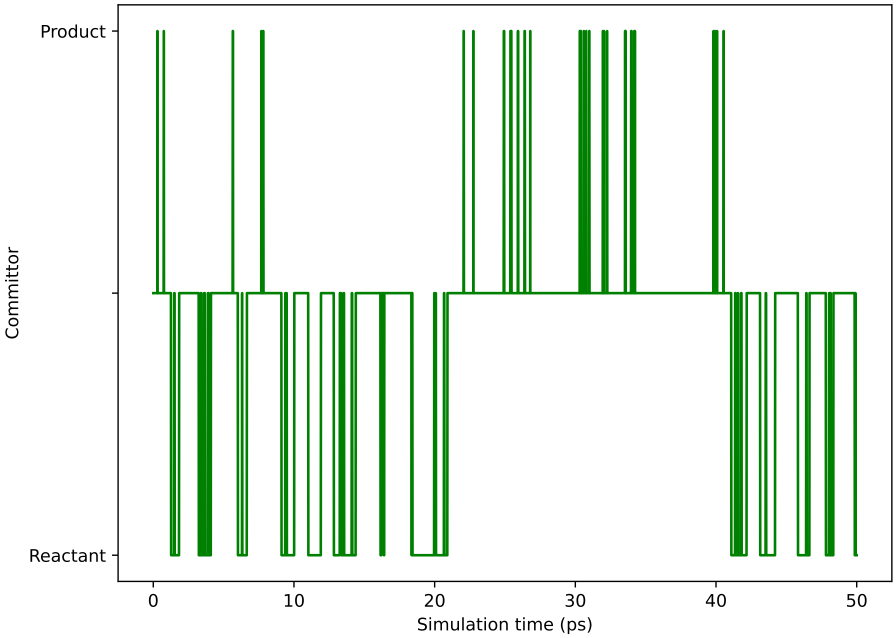

Figure S8: Committor profile for azaphilic addition for Phenyl derivative in DCM solution using MetaD and bond distance as a CV.

The existence of the local minima in FESs has been assessed using committor analysis. Bond distances were used as descriptors for the pre-formation of adduct between *s-tetrazine* and silyl-enol ether. In this assessment, C−C and C−N bond distances were used for iEDDA and azaphilic addition reactions, respectively. The committor profiles are shown in Figures S7 and S8. The structure in the trajectory committed to a basin spanned by bond distance between 1.85 Å and 1.89 Å and between 3.7 Å and 4.2 Å is reactant state and product state, respectively.

7 References

(S1) Peter Atkins, J. K., Julio de Paula *Atkins' Physical Chemistry*, 11th ed.; Oxford University Press, 2018.

(S2) Yeh, I.-C.; Hummer, G. System-Size Dependence of Diffusion Coefficients and Viscosities from Molecular Dynamics Simulations with Periodic Boundary Conditions. *The Journal of Physical Chemistry B* **2004**, *108*, 15873–15879.

(S3) Pranami, G.; Lamm, M. H. Estimating Error in Diffusion Coefficients Derived from Molecular Dynamics Simulations. *Journal of Chemical Theory and Computation* **2015**, *11*, 4586–4592.

(S4) Laio, A.; Gervasio, F. L. Metadynamics: a method to simulate rare events and reconstruct the free energy in biophysics, chemistry and material science. *Reports on Progress in Physics* **2008**, *71*, 126601.

(S5) Barducci, A.; Bonomi, M.; Parrinello, M. Metadynamics. *WIREs Computational Molecular Science* **2011**, *1*, 826–843.
